# Kinetic Analysis of Irreversible Covalent Enzyme Inhibitors and Its Use in Drug Design

**DOI:** 10.3390/ijms27083383

**Published:** 2026-04-09

**Authors:** Jean Chaudière

**Affiliations:** CNRS, IMS, UMR 5218, University of Bordeaux, 33405 Talence, France; jean.chaudiere@u-bordeaux.fr

**Keywords:** irreversible inhibition, enzyme inactivation, kinetic mechanism, drug design, targeted covalent inhibitor, mechanism-based enzyme inhibitor, suicide substrate, partition ratio

## Abstract

Irreversible covalent enzyme inhibitors, including targeted covalent inhibitors (TCIs) and mechanism-based enzyme inhibitors (MBEIs), play an increasingly important role in drug discovery. Their pharmacological behavior is governed by intrinsic inactivation parameters, typically described by the inactivation constant K_I_, the maximal inactivation rate constant k_inact_, and their ratio k_inact_/K_I_. However, no consensus exists regarding how these parameters should be experimentally determined and interpreted, particularly in high-throughput screening environments where IC_50_ values are often used as primary descriptors. This article presents a critical survey of the kinetic methodologies employed to characterize irreversible enzyme inhibition. Continuous progress-curve analysis, discontinuous end-point assays, IC_50_-based estimation strategies, direct mass-spectrometric monitoring of covalent modification, and numerical approaches required by pre-incubation protocols are examined and compared. Attention is given to the statistical robustness of parameter estimation under realistic experimental error, including bootstrap-based uncertainty analysis. For mechanism-based enzyme inhibitors, the kinetic consequences of branching between productive turnover and irreversible inactivation are analyzed, and limitations of classical half-life-based linearization methods are discussed. Intrinsic inactivation parameters are distinguished from protocol-dependent observables, and experimental conditions that may compromise reliable parameter extraction are identified. The objective is to clarify how irreversible inhibitors should be kinetically characterized when the goal is mechanistic understanding and rational drug design. By bridging classical enzymology with current discovery practices, this review provides practical guidance on what experimental data can legitimately support and where caution is required.

## 1. Introduction

The use of covalent inhibitors in therapy has a rather long history, since aspirin and penicillin were used for many years before their enzyme targets and their mechanism of action were discovered [1]. Their considerable development in the last two decades, as well as the ever-increasing underlying creativity, have been discussed in several recent reviews [1,2,3,4,5,6]. Such a resurgence of irreversible covalent inhibitors in drug discovery is best illustrated by the clinical success of blockbusters like Ibrutinib (targeting BTK) which revolutionized the treatment of chronic lymphoid leukemia, or Osimertinib (targeting tyrosine kinase of EGFR) which had a similar impact in the treatment of metastatic non-small cell lung cancer [7].

We will discuss the kinetic properties of two distinct families of irreversible covalent inhibitors, that of targeted covalent inhibitors (TCIs) and that of mechanism-based enzyme inhibitors (MBEIs).

TCIs bear an electrophilic warhead that can make a covalent bond with a nucleophilic amino-acid sidechain of the targeted active site. In drug design, enzyme selectivity is mandatory, and this is why this warhead should be as moderately electrophilic as possible, and appropriately positioned on a highly specific non-covalent ligand to ensure high effective molarities of electrophilic and nucleophilic reactants [7].

Depending on the selected warhead, TCI may be reversible or irreversible. In therapy, the expected advantage of an irreversible TCI is its infinite residence time which should maintain therapeutic effects long after the inhibitor has been cleared from circulation and up to new enzyme biosynthesis. It is outside the scope of this review to compare potential advantages and constraints of reversible or irreversible TCI.

MBEIs do not bear an electrophilic warhead, which is a major advantage in terms of systemic toxicity. They are transformed during a fraction of the catalytic turnovers into a reactive species which traps and blocks the active site [8,9,10]. Speaking of MBEI implies that the catalytic mechanism of the enzyme is involved in the “electrophilic activation” of the inhibitor. An MBEI, which is in part transformed by its target enzyme into a product, may be called a suicide substrate. In the kinetic analyses of Section 2 and Section 3, we will consider that TCI and MBEI interact specifically with the enzyme active site. This mechanistic restriction avoids the broader or sometime ambiguous terminology used in the literature.

Most of the tools which are used to study the kinetics of fast and reversible enzyme inhibition cannot be used with irreversible inhibition because there is no steady state anymore: inhibition is time-dependent. Important reviews have been published on the kinetics of irreversible enzyme inhibition in recent years [11,12,13,14,15,16,17].

Despite decades of enzymological research, there is no consensus regarding how irreversible inhibitors should be characterized experimentally. IC_50_ values remain the most frequently reported descriptors in high-throughput screening, although they are intrinsically time-dependent and protocol-dependent for irreversible mechanisms. Moreover, historical linearization strategies and half-life-based methods are still occasionally applied under experimental conditions that do not satisfy their underlying assumptions. As a result, reported parameters may lack mechanistic meaning or statistical robustness.

Irreversible inhibition must be distinguished from enzyme denaturation or from very tight but reversible binding. In most pharmacologically relevant cases, inactivation proceeds through the following two-step mechanism: a rapid reversible association step, characterized by an inhibition constant K_I_, followed by covalent bond formation with rate constant k_inact_. K_I_ is not a simple dissociation constant because EI is not a reversible dead-complex, but a reversible intermediate that precedes the dead-end covalent complex. For mechanism-based enzyme inhibitors, an additional branching step competes between productive turnover and irreversible inactivation, introducing the partition ratio r as a key parameter. These intrinsic kinetic constants are mechanistically meaningful and can, in principle, be related to selectivity and drug efficacy. However, their reliable estimation requires appropriate experimental design and data analysis.

The objective of this article is not to provide a general overview of covalent inhibitors, but to critically examine the kinetic methodologies used to characterize irreversible enzyme inhibition. We compare continuous progress-curve analysis, discontinuous end-point assays, IC_50_-based estimation procedures, direct mass-spectrometric monitoring of covalent modification, and numerical approaches required by automated pre-incubation formats. Attention is given to the robustness of parameter estimation under realistic experimental error, including bootstrap-based uncertainty analysis [18,19,20].

By explicitly distinguishing intrinsic inactivation parameters from protocol-dependent observables, and by identifying experimental conditions under which parameter extraction becomes unreliable, this review aims to clarify how irreversible inhibitors should be analyzed when the goal is rational drug design rather than descriptive reporting. The perspective adopted here is constructive: to bridge classical enzymology with current high-throughput discovery practices, and to provide practical guidance on what experimental data can legitimately support and what they cannot. In several areas, commonly used approaches are shown to be statistically inefficient or to rely on restrictive assumptions that are rarely satisfied in practice.

Section 2 and Section 3 examine kinetic methodologies for targeted covalent inhibitors and mechanism-based inhibitors, respectively. Within each, continuous, discontinuous, and automated assay formats are compared. Emphasis is placed on robustness of parameter estimation rather than mathematical derivation alone.

## 2. Irreversible Inhibition by Targeted Covalent Inhibitors (TCIs)

**Scheme 1:**
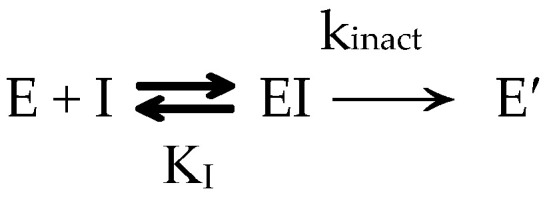
Classical Kitz-Wilson mechanism.

With TCI, irreversible inhibition develops in the absence of catalytic cycle, which means that an irreversible bond is made between the enzyme active site and the inhibitor, independently from the transformation of substrate into product. The hypothesis of specific inactivation/irreversible inhibition which is usually formulated [21] is that the first binding step which is fast leads to a reversible enzyme–inhibitor complex which then decays to an irreversibly inactivated species E′, as shown in Scheme 1:

The physical relevance of separating non-covalent recognition from the chemical step was recently validated by Ghaby and Roux [22] who used molecular dynamics and kinetic modeling to show that the overall inactivation efficiency is governed by specific protein conformations that facilitate the chemical reaction.

In the reversible portion of the mechanism, I usually behaves as competitive inhibitor of a unique substrate, or of one of the substrates of a multi-substrate enzyme. If site specificity does not rely on the production of an intermediary complex EI, it can only be due to chemical reactivity with one amino-acid sidechain of the active site. Such a covalent inhibitor is unlikely to be developed as a drug because there will be other enzymes that will be affected in vivo, but it may provide important information on the reactivity of the active site of the purified enzyme and serve as a starting basis for a more specific drug design strategy. We will focus our discussion on TCI, for which the binding specificity relies on a first reversible step, and we will name (E_0_) the initial concentration of active enzyme.

### 2.1. Demonstration of Time-Dependent Enzyme Inactivation

In a mechanism of irreversible enzyme inhibition such as that of Kitz and Wilson (Scheme 1), we have (E) + (EI) + (E′) = (E_act_) + (E′), where (E_act_) is the residual concentration of active enzyme. Therefore, we can write:

d(E′)/dt = −d(E_act_)/dt = k_inact_.(EI) (1)

If we consider that I is a fast reversible inhibitor, we have:

(EI) = (E_act_)/[1 + K_I_/(I)] (2)

Finally,

−d(E_act_)/dt = k_inact_.(E_act_)/[1 + K_I_/(I)] (3)

Which upon integration gives:

Ln[(E_act_)/(E_0_)] = −k_inact_.t/[1 + K_I_/(I)] (4)

This is equivalent to:

Ln[(E_act_)/(E_0_)] = −k_obs_.t (5a)

with k_obs_ = k_inact_/[1 + K_I_/(I)] (5b)

Therefore, the easiest way to demonstrate that time-dependent inhibition is first order is to measure the residual activity (E_act_) and plot its logarithm as a function of time. Enzyme activity may be measured with a probe substrate, on a large dilution of the reactional medium to ensure that inhibition can be neglected during the measurement.

**Figure 1:**
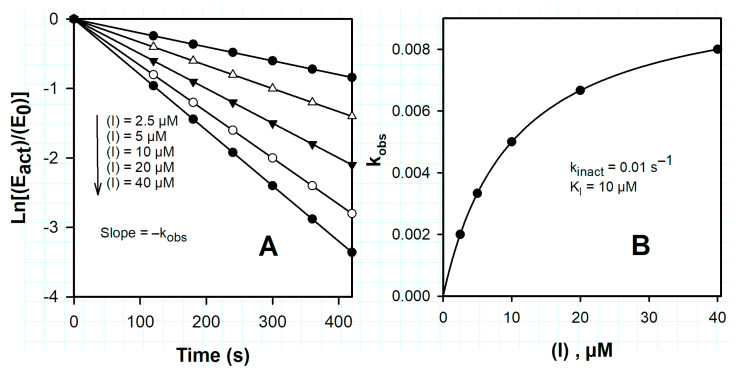
Logarithmic plot used to demonstrate first-order inactivation and to estimate k_obs_ and inactivation parameters. (**A**) kinetics of enzyme modification are simulated using (E_0_) = 10^−3^ µM, K_I_ = 10 µM and k_inact_ = 0.01 s^−1^; from bottom to top, concentrations of inactivator used are 2.5, 5, 10, 20, and 40 µM; for each (I) value, the slope of the straight line obtained by linear adjustment to Equation (5a) gives k_obs_. (**B**) non-linear adjustment of K_I_ and k_inact_ is obtained from the resulting k_obs_ = ƒ(I), Equation (5b). This gives k_inact_ = 0.0100 ± 0.0005 s^−1^ and K_I_ = 10 ± 0.26 µM, based on the estimation distribution of 100 bootstrap replicates in which 5% Gaussian errors were applied to each activity measurement.

This gives the linear plots of Figure 1A for each concentration (I). k_obs_ can then be plotted as a function of (I), as shown in Figure 1B, and if the profile confirms hyperbolic saturation, k_inact_ and K_I_ can be estimated by non-linear adjustment to Equation (5b).

The double reverse plot of 1/k_obs_ = ƒ[1/(I)] is often used but should be avoided, because it leads to implicit weighting of data that decreases the precision of estimations. For each concentration of inhibitor, the enzyme half-life t_1/2_ is equal to ln2/k_obs_.

Simulated “perfect” data cannot be used to assess the robustness of the method. Here, 100 bootstrap replicates assuming a 5% error on activity measurements gave final estimations (see Supplementary Materials S1) very close to the true values with small standard deviations. There is a small error on K_I_, while k_inact_ is indistinguishable from the expected value. This suggests that estimation improvements could be obtained by increasing the time-points or adding one smaller concentration (I) < K_I_.

#### Significance of In Vitro Measurements of K_I_ and k_inact_

In vitro kinetic experiments give the first information that may be compatible or incompatible with the pharmacokinetic profiles that are looked for in a physio-pathological situation. However, the results that will be observed in vivo will rely on additional factors which may be critical, such as metabolic degradation, protein resynthesis rate and xenobiotic export systems. The lack of strict enzyme specificity and the existence of drug–drug interactions may also be discovered at a later stage.

The inactivation constant K_I_ is equal to the concentration of inhibitor for which k_obs_ is equal to k_inact_/2.

The ratio k_inact_/K_I_ gives the apparent second-order rate constant for covalent bond formation. Since it is a second-order rate constant, it cannot exceed the diffusion limit, like the ratio k_cat_/K_M_ [23]. This limit is lower for a protein/ligand couple than that observed for two small molecules, and it depends on the size and accessibility of the protein, as well as on medium viscosity, but it is probably close to 10^6^–10^8^ s^−1^. This means that it is unlikely that the reactivity of a tight-binding inhibitor could be markedly improved by increasing its k_inact_. As a rule, however, we should consider that a specific inhibitor will require much less inhibitor for covalent inhibition than a non-specific inhibitor, because its K_I_ is much lower.

Measurement of k_inact_ should help to design inhibitors that take full advantage of the electrophilicity of the reactive group.

Selectivity gives an indication of the relationship between inhibitor concentration and the observed rate of covalent bond formation at that concentration. Depending on inhibitor concentration, this is k_inact_ or the ratio k_inact_/K_I_ which reflects selectivity. From Equation (3), we can say that for inhibitor concentrations much smaller than K_I_, the k_inact_/K_I_ ratio reflects selectivity, whereas at inhibitor concentrations much higher than K_I_, this is k_inact_ which reflects selectivity.

A good example of appropriate use of the ratio k_inact_/K_I_ is that of Ibrutinib, a major drug used as inhibitor of Bruton’s tyrosine kinase (BTK). Optimization of this ratio was crucial to obtain full inactivation of BTK at clinical doses that minimized systemic exposure [24].

Whenever possible, measuring such ratio on live cells may provide information that complement in vitro measurements. Shirley et al. [25] succeeded to do that by developing a gel-based, end-point competition assay to estimate k_inact_/K_I_ values for β-lactams as inactivators of penicillin-binding proteins.

However, individual estimations of K_I_ and k_inact_ do provide useful information. This is illustrated by the development of Afatinib, an EGFR covalent inhibitor with higher initial affinity for EGFR than inhibitors of previous generations. This lowered the necessary therapeutic concentrations and therefore the risk of reaction of its electrophilic warhead with other proteins [26].

IC_50_ values are invariably used in high-throughput drug screening, but they do not provide intrinsic mechanistic information for irreversible inhibitors, contrary to K_I_ and k_inact_. IC_50_ values obtained in different experimental set-ups cannot be compared. They depend on the selected end-point measurement (product concentration versus enzyme activity), are time-dependent, and also depend on initial concentrations of all actors (enzyme, inhibitor, and competitive substrate). Moreover, any IC_50_ value within 5–10-fold of the target protein concentration should be viewed with caution. The lowest measurable IC_50_ in a biochemical assay is one-half the target protein concentration [17]. Therefore, if one uses 10 nM enzyme with two inhibitors A and B which have the same K_I_ = 10 nM, but k_inact_(A) = 10^6^ s^−1^ and k_inact_(B) = 10^5^ s^−1^ respectively, they will have the same apparent IC_50_ of 5 nM, which does not reflect their 10-fold difference in k_inact_. Despite these limitations, IC_50_ values are still largely used in the pharmaceutical industry because their gathering is less resource- and time-demanding than that of k_inact_ and K_I_, and because they can be automated in high-throughput screening. A linear correlation between IC_50_ values and 1/(k_inact_/K_I_) has been reported for potential inactivators of JAK3 [27], based on the equation developed by Maurer et al. [28], but outliers exist, and the generalization of this equation is questionable.

The development of Nirmatrelvir (the active component of Paxlovid used in the treatment of SARS-CoV-2) provides a contemporary illustration of the necessity for rigorous kinetic profiling in drug design. This TCI targets the main protease of SARS-CoV-2 by forming a covalent adduct with its catalytic cysteine. While early candidates often used more reactive electrophiles, Nirmatrelvir was optimized to balance a reasonably fast rate of inactivation with a high degree of specificity [29]. Clearly, this optimization could not have been obtained from IC_50_ alone.

It should be underlined, however, that once reliable kinetic parameters have been estimated in vitro, they can be misused in the process of lead selection if other kinetic parameters have not been estimated in vivo, among which drug and target respective turnovers are critical.

### 2.2. Direct Monitoring of Enzyme Covalent Modification by Mass Spectrometry in the Absence of Substrate

Here, the covalent modification of enzyme is monitored as a function of time, for distinct concentrations of irreversible inhibitor (I) >> (E_0_). This method provides the most straightforward kinetic analysis because it does not depend on substrate concentration, but it requires an expensive and sophisticated set-up. It was developed by Campuzano et al. [30] with a solid-phase extraction-based time-of-flight mass spectrometer system used in combination with dedicated software. The authors report an analysis time of approximately 20 s per sample, including sample loading and desalting. The fraction of covalently modified protein is automatically determined without need for manual inspection of individual spectra. Another set-up based on Acoustic Ejection Mass Spectrometry was described more recently [31]. It is faster, since it takes approximately 1 s per sample, and it could screen approximately 1.5 million compounds in 33 days of continuous work.

From Equation (5), we can write that the concentration of modified enzyme (E′) is an exponential function of time.

(E′) = (E_0_).(1 − exp(−k_obs_.t)) (6a)

with k_obs_ = k_inact_.(I)/[(I) + K_I_] (6b)

k_obs_ is a hyperbolic function of (I) which tends to k_inact_.(I)/K_I_ when (I) << K_I_.

**Figure 2:**
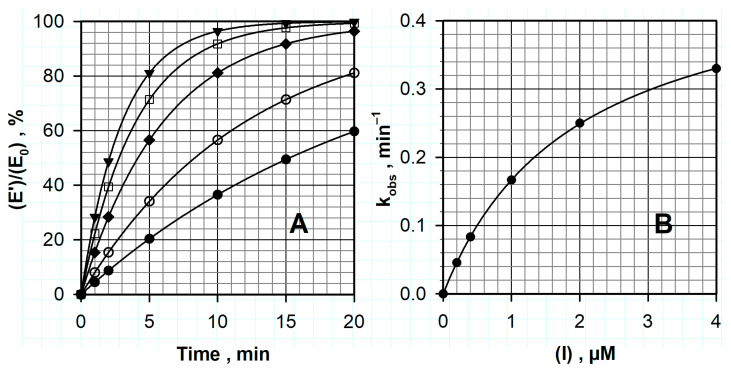
Direct monitoring of enzyme covalent modification. (**A**) kinetics of enzyme modification are simulated using (E_0_) = 2 10^−3^ µM, K_I_ = 2 µM and k_inact_ = 0.5 min^−1^; from bottom to top, concentrations of inactivator used are 0.2, 0.4, 1, 2, and 4 µM; continuous lines are obtained by non-linear adjustment of data to Equation (6a), which gives k_obs_ for each value of (I). (**B**) non-linear adjustment of the resulting k_obs_ = ƒ(I) to Equation (6b) gives k_inact_ = 0.504 ± 0.034 min^−1^ and K_I_ = 2.043 ± 0.299 µM.

Typical graphs obtained for enzyme modification as a function of time are shown in Figure 2. In Figure 2A, non-linear adjustment of k_obs_ to Equation (6a) gives an estimation of k_obs_ for each value of (I), and in Figure 2B, non-linear regression of the resulting k_obs_ = ƒ(I) to Equation (6b) gives estimations of k_inact_ and K_I_. To assess the robustness of the method, one should make reasonable assumptions on the type of errors that should apply. Here, parameter uncertainty was assessed by bootstrap resampling (100 replicates) using a combined error model consistent with MS measurements, including both point-wise (3%) and curve-wise (3%) variability (see Supplementary Materials S2). Progress curves were refitted in each replicate to estimate k_obs_, k_inact_ and K_I_.

This direct monitoring of enzyme modification is certainly ideal to screen a very large bank of compounds and then select a few of them to get more detailed information, but it only monitors the kinetics of covalent modification of the enzyme without monitoring inactivation per se. Once some candidates have been selected, one can determine binding stoichiometry and confirm sites of modification using enzymatic cleavage or top-down MS/MS [32,33]. The main limitations of MS-based progress curves are that they require expensive and highly specialized instrumentation, plus careful sample preparation. The impossibility to work with (I) >> K_I_ frequently results in the estimation of K_I_/k_inact_ only. They also may be affected by experimental artifacts which should be considered when interpreting results.

In principle, if MS is performed on intact proteins, it may be used to obtain k_inact_/K_I_ values with a full dose–response time-course (DRTC) experiment. This involves a two-dimensional matrix varying time on one axis and dose on the other, which is time-consuming and labor-intensive. Recently, Jeon et al. [34] described a new MS-based method, the diagonal dose–response time-course (dDRTC), that enables accurate determination of k_inact_/K_I_ values with eight-fold increased throughput and minimal protein consumption.

### 2.3. Monitoring Production Curves

**Scheme 2:**
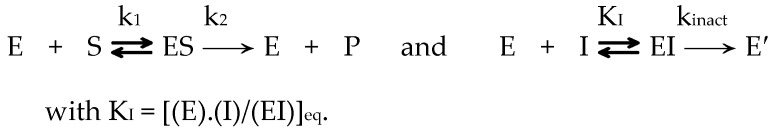
Concomitant production and inactivation mechanisms.

For a mechanism involving a single intermediary complex EI, the simplest Scheme 2 is

Since the total concentration of active enzyme decreases with time due to inactivation, (E_0_) must be replaced at each time t by the residual concentration of active enzyme (E_0_) − (E′) in the rate equation:

(E_act_) = (E_0_) − (E′) = (E_0_).exp(−k_obs_.t) (7)

This equation is similar to Equation (6a), and k_obs_ is again a function of k_inact_ and K_I_, but here, we have a substrate S which is in competition with the inhibitor I for the active site, which means that we should use K_I(app)_ = K_I_.(1 + (S)/K_M_) instead of K_I_:

(8) $$k_{\mathrm{obs}}=\frac{k_{\mathrm{inact}}\cdot \left(I\right)}{\left(I\right)+K_{I}\cdot \left(1+\frac{\left(S\right)}{K_{M}}\right)}$$

If (I_0_) >> (E_0_), the final rate of product formation is equal to zero, before complete consumption of (S), and the production curves are then described by the following exponential function:

(9a) $${\left(P\right)}_{\left(I\right),t}=\frac{V_{I}}{k_{\mathrm{obs}}}\cdot \left[1-\exp\left(-k_{\mathrm{obs}}\cdot t\right)\right]$$

with

(9b) $$V_{I}=\frac{k_{\mathrm{cat}}\cdot \left(S\right)\cdot \left(E_{0}\right)}{\left(S\right)+K_{M}\cdot \left(1+\frac{\left(I\right)}{K_{I}}\right)}$$

Here, enzyme inactivation is monitored indirectly through its effect on the rate of product formation.

In practice, initial rates should be measured over the first 10% of substrate consumption (pseudo-steady-state constraints), and it is difficult to make accurate rate estimations because the enzyme is rapidly depleted. Therefore, it is commonly preferred to replace the amplitude V_I_/k_obs_ by the final product concentration (P)_∞_:

(P)_(I),t_ = (P)_∞._(1 − exp(−k_obs_.t)) (10)

Non-linear fitting of each reaction curve to this exponential function gives k_obs_ and (P)_∞_ for each inhibitor concentration.

As previously done in Figure 1 and Figure 2, k_obs_ values can be plotted as a function of inhibitor concentration (I_0_). Again, non-linear adjustment to the expected hyperbolic function should give k_inact_ and K_I(app)_, with K_I(app)_ = K_I_.[1 + (S)/K_M_], from which K_I_ is obtained.

**Figure 3:**
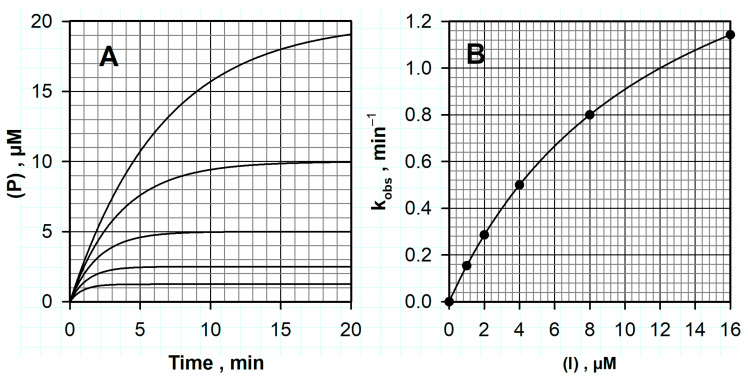
Production curves obtained for several substrate concentrations. (**A**) production curves were simulated from Equations (2)–(5), using (E_0_) = 10^−3^ µM, k_cat_ = 4.10^3^ min^−1^, (S) = 200 µM, K_M_ = 40 µM, K_I_ = 2 µM and k_inact_ = 2 min^−1^; from top to bottom, concentrations of inactivator used are 1, 2, 4, 8 and 16 µM; continuous lines are theoretical curves obtained from Equation (9), and k_obs_ is obtained for each curve by non-linear adjustment. In (**B**) based on bootstrap replicates and error assumptions described in the text, non-linear adjustment of the resulting k_obs_ = ƒ(I) to Equation (8) gives k_inact_ = 2.005 ± 0.008 min^−1^ and K_I_ = 12.0062/(1 + 200/40) = 2.010 ± 0.126 µM.

This is illustrated in Figure 3 where the uncertainty on each kinetic parameter was assessed by bootstrap resampling of simulated progress curves. A combined error model was used, consisting of a 5% curve-level (correlated) variability and 1.5% point-level variability (see Supplementary Materials S3). Distributions of k_obs_, k_inact_, and K_I_, were obtained from 100 bootstrap replicates.

The slope of the initial/linear portion of Figure 3B should be equal to k_inact_/K_I_ if at least one inhibitor concentration much lower than K_I_ has been used, and this may serve as control of the individual estimations of k_inact_ and K_I_. One should underline that the non-linear regression of Figure 3B does not estimate K_I_, but only K_I(app)_, which means that any relative error on K_M_ (for the competitive substrate) will add to that of K_I_. To minimize this problem, one should first optimize the estimation of K_M_ by means of non-linear regression on initial rate data V = ƒ(S), using duplicate points on at least (S) ~ 0.25, 0.5, 1, 2 and 4 K_M_.

Another problem which is easily underestimated when non-automated measurements are used is the uncertainty about time t_0_ = 0. If one does not work with the automated and fast mixer of a dedicated instrument, it is careful to test the procedure by repeated injections of a stable chromophore to see how many seconds it takes to get a stable absorption. Alternatively, Wimalasena and Haines [35] give a useful way to “correct” the initial data by exponential extrapolation from the reliable portion of the curve. This strategy is efficient, but it unavoidably forces exponential growth that may not always reflect what happens at the start.

It should also be underlined that non-specific thermal denaturation of the enzyme can become significant when irreversible inactivation is monitored over long incubation times. This will introduce bias in the estimation of kinetic parameters, if not properly controlled. Appropriate corrections may be envisaged for progress curves, as shown in Supplementary Materials S4. The major point is that thermal denaturation may be hidden and compensated by a lowered K_I_ in the two-step approach of K_I_ and k_inact_ estimations described in Figure 3. This overestimates inhibitor affinity, unless a first-order rate constant kth of thermal deactivation/denaturation is included in a global non-linear fitting that is directly applied to production curves. To the best of my knowledge, this is usually not done. One should at least bear in mind that subtracting the loss of enzyme observed for each time point in the absence of covalent inhibitor would not make sense, because thermal inactivation is usually first order in active enzyme and markedly dependent on substrate concentration(s) which have a protective effect.

A good example of rigorous analysis of progress curves with extraction of K_I_ and k_inact_ is that of Hopper et al. [36] who compared BTK inactivators Ibrutinib and Alacabrutinib. They underline that the relative selectivity of covalent inhibitors toward different kinase targets should be assessed with both affinity and inactivation kinetics to accurately account for time-dependent effects of covalent binding, rather than using IC_50_ at fixed time.

### 2.4. Discontinuous Assays

#### 2.4.1. Discontinuous Assays After Coincubation of Substrate Plus Inhibitor

Analyzing production curves is highly recommended to monitor enzyme irreversible inactivation, but it is not uncommon that no continuous assay is available or reliable over long periods of time. In such situations, discontinuous/end-point assays may be used.

**Figure 4:**
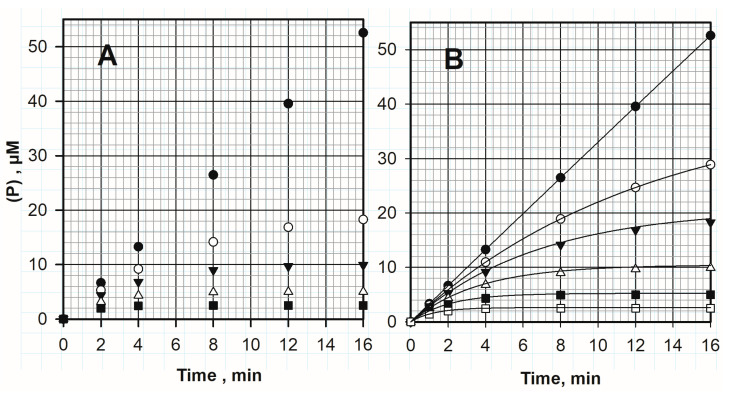
Discontinuous product assays. Simulation of experimental data is based on (E_0_) = 10^−3^ µM, k_cat_ = 4000 min^−1^, (S) = 200 µM, K_M_ = 40 µM, K_I_ = 2 µM and k_inact_ = 2 min^−1^. Inactivator I is co-incubated with enzyme and with excess substrate for several time-points. This process is repeated for increasing concentrations of I, and k_obs(app)_ is then estimated by non-linear adjustment, except that 100 bootstrap replicates assuming 5% measurement error for each point were used to evaluate parameter uncertainty. (**A**) from top to bottom, (I) = 0.01, 1, 2, 4 and 8 µM, with time sampling at 0, 2, 4, 8, 12 and 16 min. There was no significant adjustment of K_I_ and k_inact_. (**B**) (I) = 0.5 µM and t = 1 min were added to the previous grid, and this led to K_I_ = 2.27 ± 1.36 µM and k_inact_ = 2.17 ± 1.00 min^−1^.

After a given time of reaction of the enzyme with its substrate(s) and distinct concentrations of inhibitor, the reaction is quenched if necessary, and the end-point concentration of product is measured by means of absorbance, fluorescence or LC/MS assay. The analysis uses the equations that were already used with production curves, but the limited number of points and unavoidable errors on the assays will lead to larger standard errors on estimated parameters. This is shown in Figure 4, where the same parameters as that of Figure 3 were used and where 5% Gaussian errors were applied to product concentration measurements before running 100 bootstrap replicates. In Figure 4A, one gets a negative estimation for k_inact_ and an estimation of K_I_ which is very far from the true/theoretical value. Such estimations have no physical meaning and they suggest that K_I_ and k_inact_ may be highly correlated. The main message is that too much saturation at early time points, together with the 5% uncertainty, does not allow k_obs_ to be well constrained. In Figure 4B, only one earlier time (1 min) and one smaller (I_0_) concentration (0.5 µM) were added, and the estimates were K_I_ = 2.27 ± 1.36 µM and k_inact_ = 2.17 ± 1.00 min^−1^. Such values are very close to the true values of 2 µM and 2 min^−1^, even if the uncertainties are rather large.

In such discontinuous assays, it is highly desirable to avoid introduction of I before S (inhibition starts without substrate) or S before I (product formation starts before the onset of inhibition). A good compromise is to premix S and I. Another problem is the uncertainty on time t_0_ = 0, when manual assays are used. If one does not work with the automated mixer of a dedicated instrument, it is careful to test the procedure by repeated injections of a stable chromophore to see how many seconds it takes to get a stable absorption.

#### 2.4.2. Discontinuous Assays and IC_50_ Analysis

Another way to estimate K_I_ and k_inact_ is to use IC_50_ values based on measurement of product concentrations. The definition of such IC_50_ at time t is the concentration of inhibitor for which the concentration of product at time t is half that observed in the absence of inhibitor at the same time. Another definition of IC_50_ which is based on enzyme activities and not on product concentrations is commonly used in enzyme kinetics. Its use has been described [37], but has more limited use in drug screening, where the focus is on product concentrations rather than rates.

For each reaction time, plotting the concentration of product formed as a function of (I) gives a curve from which non-linear adjustments of IC_50_ and parameter h can be done, using the following equation:

(P)(t) = 100/(1 + (I)/IC_50_)^h^ (11)

where the concentration of product (P)(t) is expressed as % of control (no inhibitor).

As mentioned above, IC_50_ values as such do not provide ideal comparison criteria for series of drug candidates, but they are systematically reported in high-throughput screening databases, where K_I_ and k_inact_ values are often missing. This is the reason why Krippendorff et al. [38] looked for an equation that would establish a correspondence between such IC_50_ values and parameters K_I_ and k_inact_.

They demonstrated that the IC_50_ obtained for incubation time t was linked to inactivation parameters K_I_ and k_inact_ by the following implicit equation:

(12) $${\mathrm{IC}}_{50}\left(t\right)=K_{I}\cdot \left[1+\frac{\left(S\right)}{K_{M}}\right]\cdot \frac{2-2\cdot \exp\left(-\eta_{\mathrm{IC}50}\cdot k_{\mathrm{inact}}\cdot t\right)}{\eta_{\mathrm{IC}50}\cdot k_{\mathrm{inact}}\cdot t}$$

with

(13) $$\eta_{\mathrm{IC}50}=\frac{{\mathrm{IC}}_{50}\left(t\right)}{K_{I}\cdot \left[1+\frac{\left(S\right)}{K_{M}}\right]+{\mathrm{IC}}_{50}\left(t\right)}$$

Mader et al. [14] note that ηIC_50_.k_inact_ is equal to k_obs_ since (I)_t_ = IC_50(t)_.

**Figure 5:**
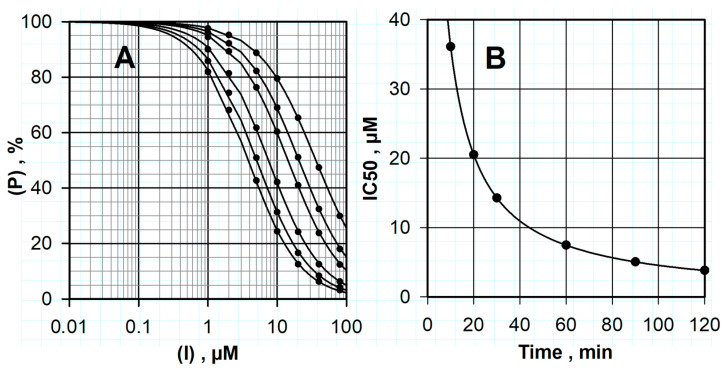
Discontinuous product assays and analysis of IC_50_ values. (**A**) IC_50_ curves were generated from product concentrations obtained with the following system: K_I_ = 20 µM, k_inact_ = 0.4 min^−1^, k_cat_ = 250 min^−1^, K_M_ = 50 µM, and (S_0_) = 250 µM. From top to bottom, IC_50_ curves were obtained for t = 10, 20, 30, 60, 90 and 120 min. For each curve, (I) = 1, 2, 5, 10, 20, 40 and 80 µM. (**B**) IC_50_ values are plotted as a function of time, from which k_obs_, K_I(app)_ and k_inact_ can be obtained by non-linear adjustment to the Krippendorf implicit Equation (12). This gives K_I(app)_ = 125.7 ± 0.75 µM and kinact = 0.429 ± 0.003 min^−1^. K_I_ is therefore equal to K_I(app)_/6 = 20.95 µM.

Equation (12) is an implicit equation, in which IC_50_ appears on both sides of the equation. This does not prevent its use to estimate k_inact_ and K_I_ by non-linear regression [39], but parameter estimations do not rely in that case on simple implementation of classical algorithms such as that of Marquardt. The non-linear adjustment used in Figure 5 can be obtained from standard graphing commercial software or from python-derived Scipy.optimize.least-squares. Fifty curves generated from a simulated set of product concentrations are shown in Figure 5A, and the non-linear least-squares adjustment of K_I_ and k_inact_ to Equation (8) is shown in Figure 5B. Precise estimations of K_I_ and k_inact_ typically require inhibitor concentrations ranging from K_I_/10 to 4 K_I_. Of course, the intermediary estimation of IC_50_ values degrades the estimations of K_I_ and k_inact_ if the amount of initial data is insufficient.

A final comment may be useful on the Krippendorff equation. In Figure 5B and in the other theoretical cases which I investigated, I found that the best function (R^2^ = 1) for non-linear adjustment was not the implicit exponential function, but a simple hyperbola IC_50_ = a.b/[(b + t)], where a and b are parameters with no obvious physical meaning. This comes from the fact that the fit is obtained from a limited number of theoretical “local” points which do not really extend to the asymptotic regions and do not have any error. This illustrates the danger of assigning any physical significance to local curvature fitting.

A good example of practical application of the Krippendorff equation is the recent study of Kopranovic and Meyer-Almes on covalent inhibitors of human histone deacetylase 8 [40]. They proposed a high-throughput strategy in which dose–response curves measured after different pre-incubation times were converted into decay curves of enzyme activity, allowing estimation of kobs values and subsequent determination of k_inact_ and K_I_.

#### 2.4.3. Discontinuous Assays After Pre-Incubation with Inhibitor

The last configuration of discontinuous assays that may be imposed is that in which the enzyme is first pre-incubated with inhibitor in the absence of substrate, after which the substrate is added for a given time. This is a sequence that is often imposed by robotized instruments designed for high-throughput drug screening. Unfortunately, such instruments are then programmed to perform discontinuous assays at a single time after addition of substrate, and one can only vary the preincubation time of enzyme with inhibitor.

During the pre-incubation period, we have (E_act_) = (E_0_).exp(−k_obs_.t_1_), where t_1_ is the preincubation time. Then, upon substrate addition, Equations (1) and (2) apply with minor modifications. Specifically, a dilution factor F_dil_ should be introduced to account for the addition of a small volume of substrate (for example, 1/1.05), (E_0_) should be replaced by (E′_0_) = F_dil_.(E_0_).exp(−k_obs_.t_1_), (I) should be replaced by (I′) = F_dil_.(I), and constant t_2_ should be introduced as time of incubation after addition of substrate, which gives the following two equations:

(14) $${\left(P\right)}_{\left(I\right),t}=\frac{V_{I}}{k_{\mathrm{obs}}^{\prime }}\cdot \left[1-\exp\left(-k_{\mathrm{obs}}^{\prime }\cdot t_{2}\right)\right]$$

(15) $$V_{I}=\frac{k_{\mathrm{cat}}\cdot \left(S\right)\cdot F_{\mathrm{dil}}\cdot \left(E_{0}\right)\cdot \exp\left(-k_{\mathrm{obs}}\cdot t_{1}\right)}{\left(S\right)+K_{M}\cdot \left(1+\frac{\left(I^{\prime }\right)}{K_{I}}\right)}$$

with k′_obs_ = k_inact_.(I′)/[K_I(app)_ + (I′)]

The problem with this experimental protocol is that we have no information on k_obs_ as well as on the values of (I) and (E_0_) at the end of the preincubation period. The biphasic nature of the assay does not enable us to find analytical solutions from Equations (14) and (15).

**Figure 6:**
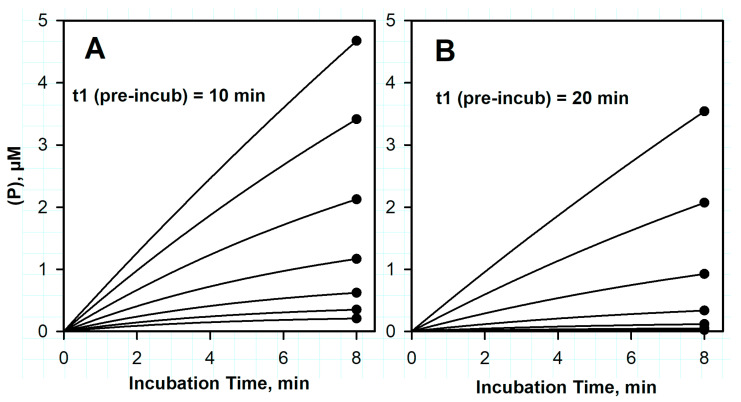
Single point product assay following preincubation with inhibitor. Data points were simulated with (E_0_) = 0.01 µM, k_cat_ = 100 min^−1^, K_M_ = 10 µM, (S) = 100 µM, Fdil = 1/1.05, K_I_ = 10 µM and k_inact_ = 0.25 min^−1^. Following a variable time of pre-incubation with inhibitor only, the substrate is added for a fixed time (t_2_ = 8 min here) and product concentration is measured. (**A**) pre-incubation time t_1_ = 10 min. (**B**) t_1_ = 20 min. Concentrations of inhibitor are (1/8, 1/4, 1/2, 1, 2, 4, 8) x K_I_. The initial grid search gave K_I_ = 9.71 µM and k_inact_ = 0.24 min^−1^. Upon running 100 bootstrap replicates based on 5% error on product measurements, one gets K_I_ = 10.01 ± 0.5 µM and k_inact_ = 0.249 ± 0.09 min^−1^.

Mader and Keillor [41] found a numeric solution by designing a simulation program (EPIC-Fit) that uses trial values for K_I_ and k_inact_ and divides pre-incubation and incubation phases into many small time-steps. At each step, the concentrations of free enzyme, inhibitor, substrate and product are updated. The program uses standard differential equation for competitive reversible inhibition, and it predicts the end-point signal for all IC_50_ curves at all pre-incubation times. A global least-squares fit then extracts a single best-fit pair of K_I_ and k_inact_. I assume that this program is mainly used on pre-selected drug candidates, based on IC_50_ values. As shown in Figure 6, I built a similar program (see Supplementary Materials S5) which does estimate K_I_ and k_inact_ with good precision, provided that the simple irreversible inhibition model is valid. Of course, it cannot extract information that is not in the original data, and it is only as valid as the kinetic model it assumes (especially no reversibility and no multiple binding modes).

## 3. Mechanism-Based Enzyme Inhibitors/Suicide Substrates

This type of inactivator simultaneously behaves as a substrate and as an irreversible inhibitor. It forms a Michaelian complex with the enzyme active site and can then follow two transformation pathways which are in competition, one which leads to product and the other one which leads to an electrophilic species that irreversibly reacts with the active site.

The concept of suicide substrate was largely developed between 1970 and 1990 [8,42], but the development of suicide substrates as drugs was slower than that of classical TCI. Examples of MBEI/suicide substrates which are currently used as drugs include, among others, penicillin and many other β-lactam antibiotics which inhibit enzymes essential for bacterial wall synthesis [43,44,45,46], clavulanate as β-lactamase inhibitor [47], exemestane used as aromatase inhibitor in the treatment of breast cancer [39,48], and ritonavir, an inhibitor of CYP3A4, used in combination with nirmatrelvir, an inhibitor of SARS-CoV-2 main protease, in the preventive treatment of COVID-19 complications [49,50].

The main advantage of MBEI/suicide substrates as potential drugs is that they do not contain an electrophilic warhead. Instead, the active site of the target enzyme is responsible for its production, which is ideal to minimize toxic effects due to partial enzyme specificity. Of course, the problem of discrimination between isoenzymes may add a lot of complexity to the structural design of pharmacologically useful suicide substrates, but this is true with any drug design strategy. More basically, one problem with the optimal design of such inactivators is that screening of pharmacological candidates requires a more sophisticated kinetic analysis.

**Figure 7:**
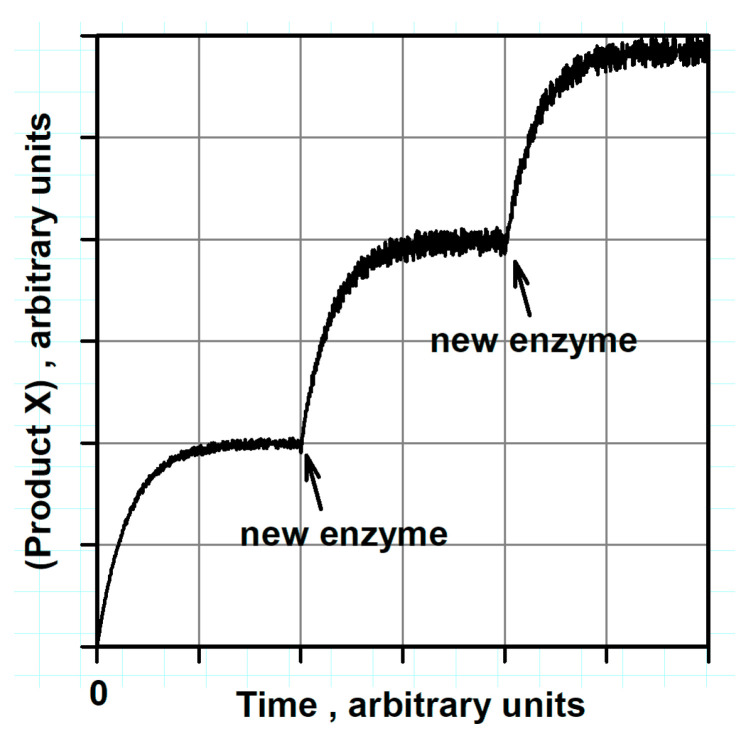
Assessing enzyme inactivation during catalytic turnover.

To prove that a substrate is a suicide substrate, the best strategy is to prove that it is transformed into product by the enzyme, and that this transformation is associated with a significant rate of inactivation of the enzyme. If we can monitor the formation of product, a profile like that of Figure 7 is a strong indication that the substrate is a suicide substrate. Here, the enzyme activity decreases to zero far before full substrate consumption, and this process can be restarted by addition of enzyme without substrate. This behavior may be undetected; however, if inactivation kinetics are considerably slower than the kinetics of product formation, or if they are so fast that no product can be detected, which is what one would look for in drug design.

Product formation stops after a jump whose amplitude is much less than the initial substrate concentration; similar jumps are subsequently observed upon addition of enzyme only.

Full characterization of the type of covalent modification of the enzyme can be performed as already discussed for other covalent inhibitors. This should bring evidence that the active site has been modified, and if the partition ratio is very low (<1), it is the best evidence that X is an MBEI, because the adduct structure should prove that it could not be produced from the original structure, but only from an electrophilic reactant derived thereof and produced by the enzyme.

### 3.1. Basic Kinetic Properties of Suicide Substrates

**Scheme 3:**
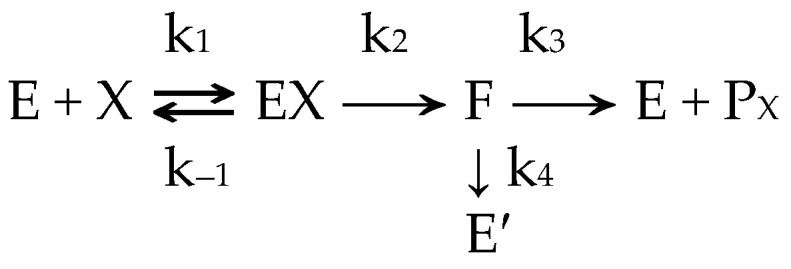
Suicide substrate mechanism.

For a one-substrate irreversible mechanism, the most common representation of this type of inhibition is that of Scheme 3 [8] which relies on the assumption that the Michaelian complex EX leads to intermediate F, which can decompose to irreversible covalent complex E′ or be transformed into product P_X_.

The first step is the reversible formation of a Michaelian complex, although the substrate is a suicide substrate written X (product P_X_), the letter S and P being used only for non-suicide/normal substrate and corresponding product, which may be added to the system as an auxiliary substrate. F is an electrophilic covalent complex, which implies that the enzyme should normally produce a similar covalent intermediate in its catalytic cycle. This means that MBEI/suicide substrates are unlikely to be designed with enzymes that have no covalent intermediate in their catalytic cycle. Many single substrate enzymes such as hydrolases contain an electrophilic covalent intermediate, and all multi-substrate enzymes which exhibit ping-pong kinetics contain at least one such covalent intermediate. By contrast, sequential mechanisms would most likely not provide covalent intermediate(s).

The covalent intermediate F is a branching point where we have two first-order parallel steps, with d(Px)/dt = k_3_(F) and d(E′)/dt = k_4_.(F).

The partition ratio r is equal to the number of molecules of suicide substrate which are transformed into product for each inactivated molecule of enzyme. It is constant, i.e., independent from enzyme and MBEI/suicide substrate concentrations:

r = k_3_/k_4_ = (Px)/(E′) (16)

Parameters K_I_ and k_inact_ which we already saw for TCI are defined as follows:

K_I_ = (k_−1_ + k_2_)(k_3_ + k_4_)/[k_1_(k_2_ + k_3_ + k_4_)] (17)

k_inact_ = k_2_k_4_/(k_2_ + k_3_ + k_4_) (18)

Here, K_I_ is the concentration of X that produces half-maximal rate of inactivation, and k_inact_ is the maximal rate constant of inactivation k_obs(max)_.

An additional parameter is necessary to describe the kinetics observed when S and X are both present:

K_iX_ = (k_−1_ + k_2_)/k_1_ (19)

Despite its analytical expression, K_iX_ is not a Michaelis constant, because the enzyme activity decreases with time. It is an inhibition constant which is ≥ K_I_ and which reflects the competitive reversible inhibition that X exerts on the probe substrate S.

The steady-state differential equations which can be derived from Scheme 3 were first provided by Tatsunami et al. [51], but analytical solutions which were derived thereof [51,52] turned out to be of limited practical use. This was due to restrictive assumptions and drastic constraints on experimental conditions (see the analysis developed in Supplementary Materials S6). Beside statistics, the main weakness of such methods is that they cannot be applied to the numerous scenarios that can be envisaged with suicide substrates, and which were clearly enumerated and analyzed by Sarmistha Dhatt [53].

Moreover, the quasi-steady-state approximation is reliable only for (X_0_) >> (E_0_). Uncertainties on the solutions increase when (X_0_) is not much larger than (E_0_), with no reliability when (E_0_)/(X_0_) ≥ 1 [54]). During enzyme inactivation by a suicide substrate, it is not always possible to work with (X_0_) >> (E_0_).

The most important point is that the outcome of an experiment [51,52] depends on the balance between the relative concentrations of enzyme and MBEI, and the value of r = (Px)/(E′).

The enzyme will be completely inactivated if (X_0_) > (r + 1).(E_0_), the final concentration of product being r.(E_0_). On the other hand, the suicide substrate will be entirely consumed before complete inactivation if (X_0_) < (r + 1).(E_0_), the final enzyme activity being (E_0_) − (X_0_)/(r + 1), and the final concentration of product being r.(X_0_)/(r + 1).

This is equivalent to saying that there will be complete inactivation if (r + 1).(E_0_)/(X_0_) < 1, whereas there will be complete suicide substrate consumption if (r + 1).(E_0_)/(X_0_) > 1.

The experimental design must take such constraints into account. In most cases, it will rely on research procedures at the bench, which are not compatible with high-throughput screening.

Experimentally, there are three options for measuring the progress of the reaction: enzyme inactivation, consumption of suicide substrate X, and appearance of product Px. The best strategy, however, is to estimate r and (K_I_, k_inact_) separately.

### 3.2. Estimation of the Partition Ratio r

To estimate the partition ratio r, the most common method consists of titrating enzyme with the MBEI. Typically, the enzyme should be incubated with increasing MBEI concentrations for at least 30 min to ensure that inactivation will not go further (this may require a longer time when dealing with a slow-binding inhibitor). Extrapolation of the initial linear portion of the curve is considered to give an intercept with the (X_0_)/(E_0_)) axis which is equal to (r + 1).

As far as the residual activity is not zero, we can write:

(E_act_) = (E_0_) − (E′) = (E_0_) − (X_0_)/[r + 1] (20)

(E_act_)/(E_0_) = 1 − [(X_0_)/(E_0_)]/[r + 1] (21)

These two equations confirm that the enzyme should be titrated—(E_act_) = 0—when (X_0_)/(E_0_) is equal to (r + 1), but they do not validate the extrapolation procedure.

To modelize the titration process, we used the following ordinary differential equations:

−d(E_act_)/dt = k_obs_(X)·(E_act_) (22)

−d(X)/dt = (r + 1)·k_obs_(X).(E_act_) (23)

d(P_x_)/dt = r·k_obs_(X).(E_act_) (24)

This system of differential equations was solved using an adaptive Runge–Kutta–Fehlberg method of order 4(5) (see Supplementary Materials S7).

**Figure 8:**
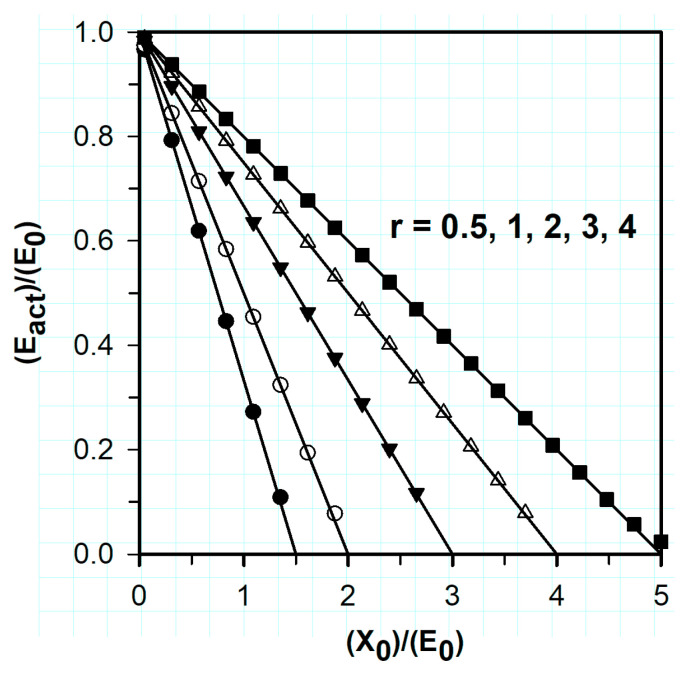
Titration plot used for estimation of the partition ratio r. Simulated titration curves show the fraction of residual active enzyme as a function of the normalized initial inhibitor concentration, evaluated at sufficiently large titration times. The endpoint curves are linear and independent of kinetic parameters; their extrapolation to complete titration yields is (X_0_)/(E_0_) = r + 1.

Figure 8 shows the fraction of residual active enzyme (E_act_)(E_0_) as a function of the normalized initial inhibitor concentration (X_0_)/(E_0_) for several values of the partition ratio r, evaluated at sufficiently large titration time.

During most of its depletion, enzyme activity decreases linearly with (X_0_), reflecting stoichiometric consumption of the enzyme. Extrapolation of the initial linear portion of the curve to complete titration does yield a ratio (X_0_)/(E_0_) which is equal to (r + 1).

When dealing with an enzyme which cannot be fully purified, such as a microsomal P_450_-dependent enzyme, the apparent residual enzyme activity may decrease to a constant minimal value which is not zero, and this value is generally used as a marker of complete titration [55,56].

One potential problem with titration curves is that of insufficient incubation times for stoichiometric titration with the inhibitor X. This is a real problem if it is a slow-binding inhibitor, which may be the case even with a very small r value. In practice, the incubation time required to obtain a reliable estimate of the partition ratio is determined by the kinetic rate of enzyme inactivation rather than by the value of r itself. If the approach to the asymptotic titration regime is approximated as a first-order relaxation with observed rate constant k_obs_.(X_0_), the time required for near-complete (99%) convergence can be estimated as t_99_ ≃ ln(100)/k_obs_.(X_0_). This corresponds to approximately 6–7 effective half-lives of the initial inactivation process. Using the classical relationship k_obs_.(X_0_) = k_inact_.(X_0_)/[K_I_ + (X_0_)], this yields an approximate lower bound t_99_ ≃ [ln(100)/k_inact_].[1 + K_I_/(X_0_)].

This expression highlights that the incubation time required to reach the asymptotic titration regime increases sharply when (X_0_) ≤ K_I_ if k_inact_ is small. Consequently, even for inhibitors with a small partition ratio, titration experiments may require long incubation times if the inhibitor is slow-binding. End-point measurements recorded before several effective half-lives of the inactivation process may therefore lead to systematic overestimation of r.

Another way to estimate r is to measure the stoichiometric ratio of product concentration and inactivated enzyme (Px)/(E′) using saturating concentrations of X, i.e., (X) >> K_I_. This is the most direct way to estimate r because it does not rely on any assumption, but this is not always possible since one should be able to measure (Px) and (E′) with high sensitivity and precision. A plot of (Px) = ƒ(E′) should then give a straight line with a slope equal to (r + 1). An interesting example [57] is that of covalent inhibition of isocitrate lyases by the suicide substrate 2-vinyl-D-isocitrate which is transformed into succinate and glyoxylate with a partition ratio r ~ 0.25. A very low partition ratio means that once catalytic intermediate F is produced, enzyme inactivation is much more likely than formation and release of Px. This reflects inhibitor high “efficiency”, but it does not imply that inactivation is fast. In fact, in the reversible portion of the mechanisms, 2-vinyl-D-isocitrate was shown to behave as a slow-binding inhibitor.

Despite their clinical success in the treatment of breast cancer, studies on the aromatase inhibitor exemestane [48,58] as well as on its cysteine conjugates [59] rely almost exclusively on static IC_50_ value. Gathering of detailed inactivation parameters, particularly the partition ratio r, should provide a starting basis for improved mechanism-based inhibitors.

One should underline that a suicide substrate with very low r (<0.3) will most likely be analyzed as a TCI in high-throughput screening. In that case, the first information that should lead to the conclusion that this is an MBEI and possibly a suicide substrate is the structure of the enzyme covalent adduct rather than kinetics, and it is important to have this information, because an MBEI will be more specific than a TCI.

### 3.3. Estimation of K_I_ and k_inact_ of MBEI/Suicide Substrates

The main strategy—known as the classical dilution protocol—does not fundamentally differ from that described for TCIs in Section 2.1, but the consequences of ill-checked assumptions may lead to incorrect parameter estimations and erroneous conclusions.

In this dilution method initially used by Walsh et al. [8] and recommended by Silverman [11], enzyme and cofactors are pre-incubated with different concentrations of inhibitor for several time points, followed by further incubation of a large dilution (at least 50-fold) of the reaction mixture with a normal (non-suicide) substrate to assess the degree of enzyme inactivation.

This method relies on two main assumptions. The first one is that inhibitor consumption is negligible during the pre-incubation step, which is plausible only if one can work with (X_0_) >> (E_0_) without extremely fast enzyme inactivation. Additionally, inhibitor titration is not the only source of its decrease if it is a suicide substrate. If the partition ratio is very high, it may be impossible to avoid a significant fall of inactivator concentration during the pre-incubation period, and its concentration should be corrected for each time point, if an assay is available.

The second assumption is that enzyme inactivation should be negligible during the following incubation step, which can be achieved if the concentration ratio of normal substrate over suicide substrate is high enough. This is the reason for the large dilution which follows the pre-incubation step.

Of course, this method also requires an enzyme concentration which is high enough during pre-incubation to make sure that enzyme activity will be measurable in the following incubation step.

Yang et al. [60] showed that these assumptions were very often unrealistic, and they demonstrated that this could result in considerable bias in the estimation of K_I_, k_inact_ and r. In a large survey, Ghanbari et al. [61] showed that such bias was expected in many publications. Nevertheless, when this classical dilution protocol is used, the results provide a first set of inhibition parameters from which one can check to which extent the original assumptions do not hold and should be corrected. This is generally not done.

### 3.4. Improved Three-Step-Based Protocol

To solve the problems observed with the classical dilution protocol, Yang et al. [62] proposed an experimental protocol which got rid of the assumption that (X) is invariant with time, and which also considered the reversible competitive inhibition between X and S, which had so far been neglected. This protocol relies on the kinetic analysis of three distinct processes, (i) transformation of inhibitor/suicide substrate into product, (ii) competitive inhibition by the probe substrate, and time-dependent irreversible inhibition. Their analysis used a genetic algorithm to estimate kinetic parameters from the three coupled differential equations which describe enzyme inactivation, suicide substrate consumption (enzyme trapping plus formation of product P_X_), and consumption of the probe substrate S:

(25) $$-\frac{d{\left(E\right)}_{t}}{\mathrm{dt}}=\frac{k_{\mathrm{inact}}\cdot {\left(X\right)}_{t}\cdot {\left(E\right)}_{t}}{K_{I}\cdot \left[1+\frac{{\left(S\right)}_{t}}{K_{\mathrm{MS}}}\right]+{\left(X\right)}_{t}}$$

(26) $$-\frac{d{\left(X\right)}_{t}}{\mathrm{dt}}=\frac{r\cdot k_{\mathrm{inact}}\cdot {\left(X\right)}_{t}\cdot {\left(E\right)}_{t}}{K_{I}\cdot \left[1+\frac{{\left(S\right)}_{t}}{K_{\mathrm{MS}}}\right]+{\left(X\right)}_{t}}$$

(27) $$-\frac{d{\left(S\right)}_{t}}{\mathrm{dt}}=\frac{d\left(P\right)}{\mathrm{dt}}=\frac{k_{\mathrm{cat}}\cdot {\left(S\right)}_{t}\cdot {\left(E\right)}_{t}}{K_{\mathrm{MS}}\cdot \left[1+\frac{{\left(X\right)}_{t}}{K_{\mathrm{iX}}}\right]+{\left(S\right)}_{t}}$$

This may be the most rigorous approach that can be envisaged with suicide substrates, but the problem which was not discussed in their analysis is that of the experimental feasibility (not only simulation) of the three-step sequence for all possible combinations of K_I_, k_inact_ and r. A detailed discussion of this problem is outside the scope of this review.

### 3.5. Production Curves of Multi-Substrate Enzymes upon Coincubation of Enzyme with Substrate and Inhibitor X

If r is small, monitoring production curves is unlikely to be feasible. If r is high, monitoring production curves is possible, but large concentrations of suicide substrate may be necessary to visualize enzyme irreversible inhibition. This probably explains why the monitoring of production curves has not been frequently used with suicide substrates.

**Figure 9:**
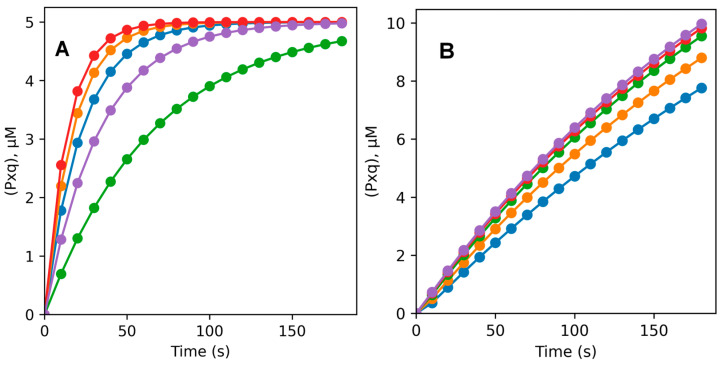
Production curves expected from suicide substrate inhibition in Bi Bi Ping–Pong mechanism. Progress curves were simulated. (**A**) (A_0_) = 240 µM; K_I_ = 10 µM, k_inact_ = 0.1 s^−1^, and r = 5; from top to bottom (X_0_) = 50, 20, 10, 5 and 2 µM. Estimations were K_I_ = 9.778 µM, k_inact_ = 0.09821 s^−1^ and r = 4.999. (**B**) (A_0_) = 100 µM; K_I_ = 20 µM, k_inact_ = 0.2 s^−1^ and r = 20; from top to bottom (X) = 20, 10, 5, 2, 1 and 0.5 µM. Estimations were K_I_ = 10.68 µM, k_inact_ = 0.1035 s^−1^ and r = 20.86.

I simulated progress curves of suicide substrate-derived product Pxq for a Bi Bi Ping–Pong enzyme in which a suicide substrate X replaces the second substrate and reacts with the modified enzyme form F. The system was simulated using coupled ordinary differential equations solved numerically (see Supplementary Materials S8). Progress curves were generated for combinations of (A_0_) and (X_0_) over 180 s. Gaussian noise (1% of maximal signal) was added to mimic experimental uncertainty. Simulated progress curves were then globally fitted by nonlinear least squares to recover K_I_, k_inact_ and r. Fits were performed directly on progress curves, without assuming single-exponential behavior or treating K_MA_ as dissociation constant. As shown in Figure 9A, when substrate-A depletion was negligible, global fitting accurately recovered all kinetic parameters. By contrast, modest substrate depletion (≤25%) produced progressive stacking of the production curves (Figure 9B), reflecting a loss of kinetic information. Under these conditions, systematic bias affected the estimation of K_I_ and k_inact_, whereas the partition ratio remained comparatively robust. Exploration of other combinations of kinetic parameters produced similar curve stacking, again with reliable estimation limited to r.

These results illustrate an intrinsic limitation of production-curve analyses when substrate depletion becomes significant. These limitations are not merely theoretical. For example, a recent study describing the covalent JNK3 inhibitor JC16I as potential drug in Parkinson disease reported estimates of K_I_ and k_inact_ from time-dependent inhibition curves [63], but without statistical analysis of the uncertainty associated with these parameters. Such situations likely reflect the limited information content of progress curves when substrate depletion affect the time course [64], making simultaneous estimation of K_I_ and k_inact_ difficult.

## 4. Concluding Remarks: Practical Implications for Drug Design

From a practical perspective, optimal selection of covalent inhibitors cannot rely on in vitro K_I_ and k_inact_ values alone but must consider their interplay with in vivo turnover rates of both the target enzyme and the inhibitor, which determine whether affinity, reactivity, or residence time are the dominant drivers of efficacy. For instance, rapidly turning-over targets require inhibitors with fast inactivation kinetics (high k_inact_), whereas slowly renewed targets may be efficiently controlled by high-affinity (low K_I_) but slower-reacting compounds. Conversely, rapid drug clearance requires fast inactivation, while long-lived inhibitors allow greater reliance on binding affinity. Recent pharmacokinetic/pharmacodynamic models explicitly combine drug-target binding kinetics with pharmacokinetics and target turnover to predict in vivo efficacy [65].

Additionally, a major decision factor is the potential existence of drug–drug interactions, frequently due to irreversible covalent inhibition of cytochrome P_450_-dependent enzymes which get rid of xenobiotics [66,67,68]. Many drugs are transformed by such enzymes into electrophilic metabolites which may inactivate the P_450_-dependent enzyme itself, thereby inducing a marked increase in half-life of some other drug. Looking at the potential irreversible inhibition of P_450_-dependent enzyme(s) is therefore essential. This requires dedicated kinetic tools [69,70], but here again, the important point is that favoring the lowest K_I_/k_inact_ among several drug candidates is unreasonable if this results in P_450_ inactivation.

These examples illustrate the need to collect reliable and meaningful kinetic parameters of enzyme inactivation, based on the principles developed in this review as follows:

First, intrinsic inactivation parameters (K_I_, k_inact_, and their ratio k_inact_/K_I_) provide mechanistically interpretable quantities that reflect reversible binding, chemical reactivity, and overall inactivation efficiency. In contrast, IC_50_ values are protocol-dependent and time-dependent observables. Although convenient for high-throughput screening, IC_50_ values cannot be directly compared across assay formats, incubation times, or enzyme concentrations. For irreversible inhibitors, ranking compounds solely on IC_50_ may obscure substantial differences in k_inact_ or K_I_, particularly when enzyme concentrations approach the IC_50_ range.

Second, the ratio k_inact_/K_I_ is often interpreted as an apparent second-order rate constant for covalent bond formation and is therefore useful for comparing compounds at low inhibitor concentrations. However, at concentrations approaching or exceeding K_I_, selectivity becomes dominated by k_inact_. Optimization strategies that focus exclusively on increasing k_inact_ without improving binding affinity may therefore fail to enhance selectivity in physiologically relevant concentration ranges.

Third, for mechanism-based enzyme inhibitors/suicide substrates, the partition ratio r is a critical determinant of pharmacological efficiency. A low r implies that few catalytic turnovers are required to inactivate the enzyme, whereas a high r indicates predominant productive turnover. Experimental protocols that do not account for incomplete inactivation or substrate depletion may lead to substantial misestimation of r and associated kinetic constants.

Finally, automated high-throughput formats often impose discontinuous or pre-incubation assay designs that restrict the information content of the data. In such cases, numerical simulation approaches may recover meaningful parameters, but only if the underlying mechanistic model is valid and experimental uncertainty is carefully considered.

From a drug discovery perspective, the key practical message is that irreversible inhibitors should be characterized using experimental designs that permit reliable estimation of intrinsic inactivation parameters, rather than relying solely on end-point potency metrics. The additional effort required for rigorous kinetic analysis is justified when irreversible binding is central to therapeutic mechanism. The development of irreversible inhibitors requires kinetic characterization strategies that are commensurate with their mechanistic complexity. Adoption of robust analytical frameworks will improve compound ranking, reduce misinterpretation, and strengthen translational reliability.

## Data Availability

No new data were created or analyzed in this study. Data sharing is not applicable to this article.

## Supplementary Materials

The following supporting information can be downloaded at https://www.mdpi.com/article/10.3390/ijms27083383/s1.

## Abbreviations

BTK	Bruton’s tyrosine kinase
EGFR	Epidermal growth factor receptor
HTS	High-throughput screening
MBEI	Mechanism-based enzyme inhibitor
MS	Mass spectrometry
TCI	Targeted covalent inhibitor
