# Supplementary material for: Kinetic Analysis of Irreversible Covalent Enzyme Inhibitors and Its Use in Drug Design
Source: Int J Mol Sci. 2026 Apr 9;27(8):3383. doi: 10.3390/ijms27083383 (PMC13116466; doi:10.3390/ijms27083383)
Supplement: Supplementary file 1 [file ijms-27-03383-s001.zip › ijms-4215122-supplementary.pdf]

International Journal of Molecular Sciences

MDPI

Supplementary Materials to:

Kinetic analysis of irreversible covalent enzyme inhibitors and its use in drug design

Jean Chaudière

SM-1. General method of error simulation and bootstrap procedures

Bootstrap-based uncertainty estimation was performed by Monte-Carlo resampling of the experimental activity time courses: for each original activity value, we generated pseudo-datasets by adding independent Gaussian noise (relative error = 5%), and repeated the full fitting workflow for each replicate (linear fit of ln(E_{act}/E_0) versus time to obtain k_{obs}, then non-linear fit of k_{obs} vs [I] to estimate k_{inact} and K_I).

Implementation used Python with NumPy for random sampling and array handling, and SciPy (scipy.stats.norm for Gaussian noise, and scipy.optimize.curve_fit, or least_squares for regression), iterating over replicates and storing fitted parameters to obtain their empirical distributions. Parameter standard deviations were taken as the sample SD across the bootstrap distributions.

SM-2. Combination of curve-wise and point-wise errors in MS-derived progress curves of enzyme inactivation

To mimic MS-derived inactivation/progress curves, uncertainty was estimated by boot-strap resampling (100 replicates) using a combined relative-error model: (i) point-wise variability (independent noise on each time point; 3% CV) and (ii) curve-wise variability (a single multiplicative factor shared by all points of a given curve; 3% CV).

Curve-wise errors were introduced because MS quantification can be globally biased for an entire data set/curve (e.g., differences in ionization efficiency, recovery, or normalization), producing systematic over- or underestimation rather than purely independent scatter.

Implementation used Python/NumPy for resampling and SciPy for regression (e.g., scipy.stats.norm for Gaussian sampling, and scipy.optimize.curve_fit/least_squares for fitting), storing fitted parameters for each replicate to compute mean ± SD. Parameter uncertainty was assessed using the same Monte-Carlo bootstrap approach as described above, in which synthetic datasets were generated by resampling the original curves with postulated relative errors. Point-wise errors correspond to independent Gaussian noise applied to each measurement, while curve-wise errors were implemented as a single multiplicative factor applied to all points of a given curve, reflecting global over- or underestimation typical of mass-spectrometry runs (MS quantification can be globally biased for an entire run/curve, due to differences in ionization efficiency, recovery, or normalization. Both error components were combined within each bootstrap replicate, and standard deviations were obtained from the resulting parameter distributions across replicates.

SM-3. Curve-wise errors in absorbance-derived progress curves of product formation

Parameter uncertainty was estimated by bootstrap resampling using the Monte-Carlo procedure described above. In addition to point-wise relative errors, a curve-wise multiplicative error was included to account for systematic effects affecting an entire progress curve, such as pipetting inaccuracies, uncertainty in the effective time zero, or small global offsets in absorbance normalization

SM-4. Corrections required for non-specific thermal denaturation over long incubation times

Here, I take the example of a one substrate enzyme, typically a hydrolase (protease, phosphatase, esterase, lipase etc.). Spontaneous/thermal enzyme deactivation during long-course progress curves was modeled as a first-order process. To maintain model parsimony while reflecting biochemical reality, I assumed that ligand binding (either substrate or covalent inhibitor) significantly stabilizes the enzyme's tertiary structure, thereby providing protection against thermal unfolding. Therefore, the deactivation rate is primarily driven by the free enzyme fraction. The apparent deactivation rate constant at any time t can then be defined as $k_{th(app)} = k_{th}.[K_M/(K_M + (S)]$

where k_{th} is the first-order rate constant for deactivation of the free enzyme.

If a production curve is obtained for $(S_0) >> K_M$, the enzyme exists predominantly in its ligand-bound forms (ES, E-acylated and EI). This approach accounts for the substrate protective effects with a progressive depletion of substrate which leads to a gradual increase in the free enzyme fraction, while covalent inactivation of the enzyme by the TCI decreases the amount of active enzyme. This phenomenon cannot be captured by simple baseline subtraction or by any simple control experiment.

K_I, k_{inact} and k_{th} should therefore be estimated by non-linear adjustment to the following equation:

$$(P)_t = (V_i/k_{obs}).[1 - \exp(-k_{obs}.t)] \qquad \text{(SM4-1)}$$

$$\text{with } V_i = k_{cat}.(E_0).(S)/[K_M + (S)], \text{ where } (S) = (S_0) - (P) \qquad \text{(SM4-2)}$$

$$\text{and } k_{obs} = \frac{k_{inact}.(I)}{(I) + K_I.[1 + (S)/K_M]} + \frac{k_{th}.K_M}{K_M + (S)} \qquad \text{(SM4-3)}$$

As shown in Figures SM-4, introducing $k_{th} = 2.10^{-4}\ s^{-1}$ in the modeling process (progress curves obtained by numerical integration) and keeping all concentrations and parameters used in figure 3 (section 2 of the main text), one may compare the estimations of K_I and k_{inact} with or without taking thermal deactivation into account.

If thermal inactivation is neglected, the errors on K_I and k_{inact} are respectively 18% and 11%, compared with true values By contrast, if thermal denaturation is incorporated into global non-linear fitting to production curves, the errors on K_I and k_{inact} are negligible.

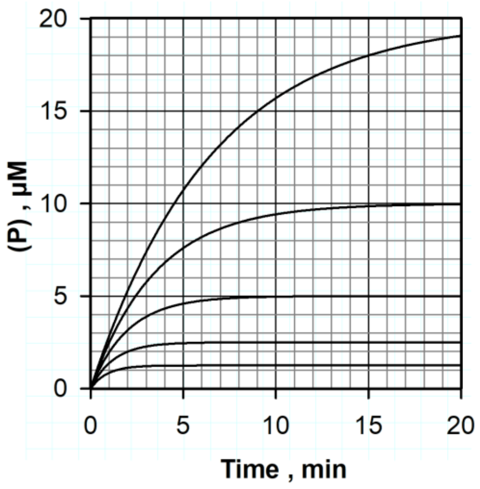

Figure SM-4. Including thermal deactivation/denaturation in the estimation of K_I and k_{inact}.

All concentrations and kinetic parameters are those of figure 3 (in the main text). Progress curves were obtained by numerical integration, using $k_{th} \sim 2.10^{-4}\ s^{-1}$. If k_{th} is neglected, a global non-linear fit gives K_I = 1,64 ± 0.08 µM (actual value = 2 µM) and k_{inact} = 1.78 ± 0.06 min^{-1} (actual value = 2 min^{-1}). If the model of thermal denaturation is used, a global nonlinear fit gives K_I = 1.98 ± 0.03 µM, k_{inact} = 1.97 ± 0.04 min^{-1} and $k_{th.}$ = 2.1 10^{-4} ± 0.04 min^{-1} which is very close to actual values for the three parameters.

Two important points should be underlined. First, Neglecting the thermal deactivation process forces K_I and k_{inact} to re-adjust without resulting in a very poor fit. Second, the reliable estimations of these two parameters plus k_{th} requires a direct/global nonlinear fit to production curves. If K_I and k_{inact} are estimated from nonlinear fit to k_{obs} = f(I), the information is essentially lost. Overall, these observations underline the need to envisage thermal denaturation in non-linear adjustments, unless separate experiments indicate that enzyme denaturation is negligible over the time of production monitoring, and for the lowest substrate concentration that is finally reached.

Extending this reasoning to multi-substrate enzyme is more tedious but not more difficult, provided that product inhibition can be neglected in the experimental conditions selected to collect production curves..

SM-5. Program designed to estimate K_I and k_{inact} from single-point product measurement after pre-incubation of enzyme with inhibitor alone.

Parameter estimation was performed by explicit simulation of the analytical forward model describing the two-phase inactivation/dilution experiment, followed by least-squares minimization. A coarse logarithmic grid search over k_{inact} and K_I was first used to locate the global minimum of the sum of squared residuals between simulated and observed product concentrations. The parameter space was then refined locally using a denser grid centered on the coarse optimum to improve precision. All calculations were implemented in Python using NumPy for numerical evaluation and array handling.

SM-6. Equation developed by Waley (1985), and limits of its application

We start from $(E_{act}) = (E_0) – (E')$ and $r = (Px)/(E')$

If X is entirely consumed before full inactivation (r large), one can write:

$$(E')_\infty = (X_0)/[1 + r] \qquad \text{(SM6-1)}$$

and Waley shows that at any time t before full consumption of X the progress curve of enzyme inactivation is described by:

$$t = N.\ln(1 – M.z) – N'.\ln(1 – z) \qquad \text{(SM6-2)}$$

which can be rearranged for $t = t_{1/2}$:

$$(X_0).t_{1/2} = \frac{K_I.\ln(2 – M)}{k_{inact}.[1 – M]} + [\ln 2/k_{inact}].(X_0)) \qquad \text{(SM6-3)}$$

where parameters N, N', M and z are defined in table 1, and $t_{1/2}$ is the time for which z = 0.5:

$$t_{1/2} = N.\ln(2 – M) + (N' – N).\ln 2 \qquad \text{(SM6-4)}$$

Table 1. Definition of parameters used by Waley (1985)

Symbol	*Definition*	*Dimension*
**r**	k_3/k_4	–
**M**	$[r + 1].(E_0)/(X_0)$	–
$\mathbf{k_{inact}}$	$k_2.k_4/[k_2 + k_3 + k_4]$	1/time
$\mathbf{K_I}$	$[\frac{k_{-1} + k_2}{k_1}].[\frac{k_3 + k_4}{k_2 + k_3 + k_4}]$	concentration
**N**	$K_I/\{k_{inact}.[1 – M].(X_0)\}$	time
**N'**	$1/k_{inact} + N$	time
**z**	$(E')/(E_0)$	–
**u**	$(X)/(X_0)$	–

As shown in figure SM-6a, the graph of $(E')/(E_0) = f(t)$ can be used to estimate $t_{1/2}$ by interpolation.

To use equation SM6-3, one should estimate $t_{1/2}$ for different values of (X_0) while maintaining the ratio $(E_0)/(X_0)$ constant. In such conditions, M is constant, and from table 1 , N et N' only depend on (X_0).

The graph of $(X_0).t_{1/2} = f(X_0)$ is therefore a straight line (see figure 1b) whose slope and x intercept can be used to estimate k_{inact} and K_I. This is why Waley proposed an estimation strategy based on measuring the half-time of enzyme inactivation $t_{1/2}$ for a series of experiments performed at different initial suicide substrate concentrations (X_0), while maintaining a constant ratio $(X_0)/(E_0)$. This is illustrated in figure SM-6b.

My tests show that under restrictive conditions (large partition ratio r, large $(X_0)/(E_0)$, and complete inactivation), $(X_0).t_{1/2}$ does vary linearly with (X_0), allowing estimation of kinetic parameters from the slope and intercept. In practice, this requires multiple complete time-courses, accurate determination of $t_{1/2}$, sufficiently long incubations to

ensure 50% inactivation is reached, and precise control of timing, making this work experimentally demanding and not applicable to many systems

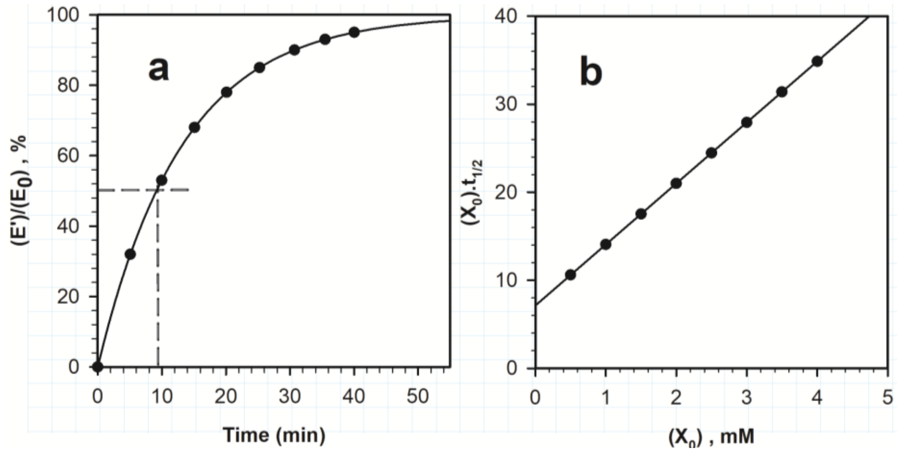

Figure SM-6. Inactivation kinetics, estimation of $t_{1/2}$ for distinct values of (X_0), and estimation of K_I, k_{inact} and r. In this example, r = 100, k_{inact} = 0.1 min^{-1} and K_I = 1 mM; the ratio $(X_0)/(E_0)$ is fixed at 1000, therefore M = 0.1. **(a):** Inactivation kinetics for (X_0) = 3.2 mM. $t_{1/2} \simeq 9.1$ min is estimated by interpolation. **(b):** Several graphs (a) obtained for different values of (X_0) are used to build the linearization graph; k_{inact} = ln2/slope; K_I = [y intercept].k_{inact}.[1 – M]/ln(2 – M).

The robustness of this strategy was evaluated using a Python-based Monte-Carlo simulation framework. Synthetic inactivation curves were generated by numerical integration of the underlying kinetic differential equations, using true parameter values. Measurement noise was introduced as relative Gaussian errors (5%) on simulated activity values, and $t_{1/2}$ values were extracted by interpolation. For each bootstrap replicate, linear regression of $(X_0).t_{1/2}$ versus (X_0) was used to estimate apparent kinetic parameters. Distributions of estimated parameters were obtained from 100 bootstrap replicates, allowing calculation of mean values, standard deviations, and failure rates (undefined $t_{1/2}$). All simulations and analyses were implemented using Python, NumPy, SciPy (solve_ivp, linregress), and standard numerical interpolation tools.
Under highly favorable conditions —large r (≥ 50), large $(X_0)/(E_0)$ (≥ 500), complete inactivation, and negligible timing uncertainty—the method gave unbiased parameter estimates, but with relatively large standard deviations compared with direct global fitting approaches. The method is statistically inefficient, because it compresses each progress curve into a single interpolated quantity ($t_{1/2}$) and amplifies experimental uncertainty through linearization. Outside such "ideal" and restrictive conditions —particularly at lower r values (r = 5), or in the presence of timing or curve-wise errors—the method did not give any reliable estimation of K_I, k_{inact} and r. One may conclude that it is experimentally heavy and not robust compared with modern methods of routine kinetic analysis.

SM-7. Simulation of titration curves and effect of insufficient incubation times.

Titration curves used to analyze the partition ratio r were generated by numerical integration of the coupled ordinary differential equations 22-24 describing enzyme inactivation, inhibitor consumption, and product formation. The system was solved using an adaptive Runge–Kutta–Fehlberg algorithm of order 4(5) (RK45), as implemented in scipy.integrate.solve ivp, with absolute and relative tolerances set to default SciPy values. To facilitate comparison across conditions, time was expressed in the dimensionless form T = $k_{obs(max)}$·t, where $k_{obs(max)}$ is the value of k_{obs} for (X) = (X_0), i.e., $k_{inact}.(X_0)/[K_I + (X_0)]$. Simulated residual activities were then used to compute apparent values of r and assess systematic errors associated with finite incubation times.

SM-8. Simulation of Bi Bi Ping-Pong system plus suicide substrate with or without first substrate consumption.

The reaction was modeled as a Bi Bi Ping-Pong mechanism in which the second substrate is replaced by a suicide substrate X. The enzyme reacts first with substrate A, generating a modified enzyme intermediate F. The latter reacts with the suicide substrate X, leading either to productive turnover (product name = Pxq) or irreversible inactivation.

The first half-reaction flux ($E + A \longrightarrow F + P$) is described by the following Michaelis form:

$V_1 = k_2.(E).(A)/[K_{MA} + (A)]$ (SM8-1) where k_2 is a first order (catalytic) rate constant.

Then the next reaction steps are:

$$F + X \underset{K_I}{\rightleftarrows} FX \qquad \text{(SM8-2)}$$

$$FX \xrightarrow{k_q} E + Pxq \qquad \text{(SM8-3)}$$

$$FX \xrightarrow{k_{inact}} E_{inact} \qquad \text{(SM8-4)}$$

We can write $k_q = r.k_{inact}$, where r is the partition ratio.

We assume rapid equilibrium binding of X to F:

$$(FX) = (F).\frac{(X)}{K_I + (X)} \qquad \text{(SM8-5)}$$

The system dynamics are defined by:

$d(E)/dt = -V_1 + V_q$ (SM8-6)

$d(F)/dt = V_1 - V_q - V_{inact}$ (SM8-7)

$d(E_{inact})/dt = V_{inact}$ (SM8-8)

$d(Pxq)/dt = V_q$ (SM8-9)

$d(A)/dt = -V_1$ (SM8-10) *A depletion is considered*

Two experimental scenarios were simulated:

(a) **constant (A)**, corresponding to negligible substrate consumption

(b) **partial A depletion**, in which substrate consumption was explicitly modeled. Numerical integration was performed using the LSODA algorithm implemented in scipy.integrate.solve_ivp.

Under constant A conditions (scenario a), the instantaneous rate of Pxq formation reduces to a classical Ping-Pong form:

$$V_q \sim \frac{(E).(A).(X).r.k_{inact}}{K_I.(A) + K_{MA}.(X) + (A).(X)} \qquad \text{(SM8-11)}$$

If we compare this equation to the classical Bi Bi Ping-Pong rate equation in the absence of product inhibition:

$$V = \frac{k_{cat}.(E_0).(A).(B)}{K_{MB}.(A) + K_{MA}.(B) + (A).(B)} \qquad \text{(SM8-12)}$$

We see that B is replaced by X, Q is replaced by PxQ, k_{cat} is replaced by $r.k_{inact}$ and K_{MB} is replaced by K_I.

Our model is therefore a time-resolved mechanistic generalization of the classical Ping-Pong equation.

Progress curves of Pxq production were generated for combinations of (A_0) and (X_0) over 180 s with sampling every 10 s. Gaussian noise (1% of maximal signal) was added to mimic experimental uncertainty on real progress curves.

In scenario b (partial A depletion), the only change in the simulation is that the program tunes constant $k_1 = k_2/K_{MA}$ to ensure that A depletion reaches 5-25% by $t_{(end)}$.

The resulting simulated progress curves were globally fitted by nonlinear least squares to recover K_I, k_{inact} and r.

Fits were performed directly on progress curves, without assuming single-exponential behavior or treating K_{MA} as dissociation constant.
